# Generation of heterozygous fibrillin-1 mutant cloned pigs from genome-edited foetal fibroblasts

**DOI:** 10.1038/srep24413

**Published:** 2016-04-14

**Authors:** Kazuhiro Umeyama, Kota Watanabe, Masahito Watanabe, Keisuke Horiuchi, Kazuaki Nakano, Masateru Kitashiro, Hitomi Matsunari, Tokuhiro Kimura, Yoshimi Arima, Oltea Sampetrean, Masaki Nagaya, Masahiro Saito, Hideyuki Saya, Kenjiro Kosaki, Hiroshi Nagashima, Morio Matsumoto

**Affiliations:** 1 Meiji University International Institute for Bio-Resource Research, Kawasaki, 214–8571, Japan; 2 Laboratory of Developmental Engineering, Department of Life Sciences, School of Agriculture, Meiji University, Kawasaki, 214–8571, Japan; 3 Department of Orthopaedic Surgery, Keio University School of Medicine, Tokyo, 160–8582, Japan; 4 Anti-aging Orthopaedic Research, Keio University School of Medicine, Tokyo, 160–8582, Japan; 5 Department of Pathology, Keio University School of Medicine, Tokyo, 160–8582, Japan; 6 Division of Gene Regulation, Institute for Advanced Medical Research, Keio University School of Medicine, Tokyo, 160–8582, Japan; 7 Division of Operative Dentistry, Department of Restorative Dentistry, Tohoku University Graduate School of Dentistry, Sendai, 980–8575, Japan; 8 Center for Medical Genetics, Keio University School of Medicine, Tokyo, 160–8582, Japan

## Abstract

Marfan syndrome (MFS) is an autosomal dominant genetic disease caused by abnormal formation of the extracellular matrix with an incidence of 1 in 3, 000 to 5, 000. Patients with Marfan syndrome experience poor quality of life caused by skeletal disorders such as scoliosis, and they are at high risk of sudden death from cardiovascular impairment. Suitable animal models of MFS are essential for conquering this intractable disease. In particular, studies employing pig models will likely provide valuable information that can be extrapolated to humans because of the physiological and anatomical similarities between the two species. Here we describe the generation of heterozygous fibrillin-1 (*FBN1*) mutant cloned pigs (+/Glu433AsnfsX98) using genome editing and somatic cell nuclear transfer technologies. The *FBN1* mutant pigs exhibited phenotypes resembling those of humans with MFS, such as scoliosis, pectus excavatum, delayed mineralization of the epiphysis and disrupted structure of elastic fibres of the aortic medial tissue. These findings indicate the value of *FBN1* mutant pigs as a model for understanding the pathogenesis of MFS and for developing treatments.

*FBN1* encodes fibrillin-1 (FBN1), which is a 350-kDa glycoprotein comprising 2,871 amino acid residues. FBN1 is the principal structural component of extracellular microfibrils^1^,^2^,^3^ and is distributed throughout the body as a connective-tissue matrix of tissues such as skin, lung, kidney, vessels, cartilage, tendon, muscle, cornea, and ciliary zonule^1^. FBN1 is intimately involved in the transforming growth factor-β (TGF-β) signalling pathway, which is essential for cell proliferation and differentiation. TGF-β1 binds to fibrillin-1 via the latent TGF-β-binding protein (LTBP) and controls signalling through this pathway by inhibiting the binding of TGF-β1 to its receptor^4^,^5^.

Marfan syndrome (MFS) (OMIM #154700) is an autosomal-dominant disorder of connective tissue caused by mutations of *FBN1*^6^,^7^. The symptoms of MFS manifest principally as cardiovascular and skeletal abnormalities. The primary cause of death of patients with MFS is progressive aortic dilation, principally in the sinus of Valsalva, leading to aortic dissection or rupture^8^,^9^. Skeletal symptoms include a tall and thin physique, long limbs, arachnodactyly, thoracic deformity (pectus carinatum, pectus excavatum), scoliosis, a high-arched palate, and chronic joint laxity. Other symptoms include ectopia lentis and myopia, skin striae atrophicae, and recurrent hernia or pneumothorax^8^,^9^.

Studies of *FBN1* mutant mice^10^,^11^,^12^,^13^,^14^ have contributed valuable information regarding the causes of MFS and for the development of treatments. However, small animals such as rodents are inadequate as models for humans to guide development of treatments involving surgical techniques to address cardiovascular or skeletal system manifestations. Therefore, large animals exhibiting a phenotype similar to the cardiovascular and skeletal manifestations of patients with MFS may contribute to the development of new treatments of patients with MFS and for gaining further insights into the pathogenesis of this intractable disease.

The application of recently developed genome editing and somatic cell cloning techniques has markedly improved the efficiency of generating pigs with gene mutations^15^,^16^,^17^,^18^,^19^,^20^,^21^,^22^,^23^,^24^. In the present study, we generated heterozygous *FBN1* mutant cloned pigs and their progeny, and showed that they developed phenotypes resembling those of patients with MFS.

## Results

### Generation of heterozygous *FBN1* mutant cell lines

**Figure 1:**
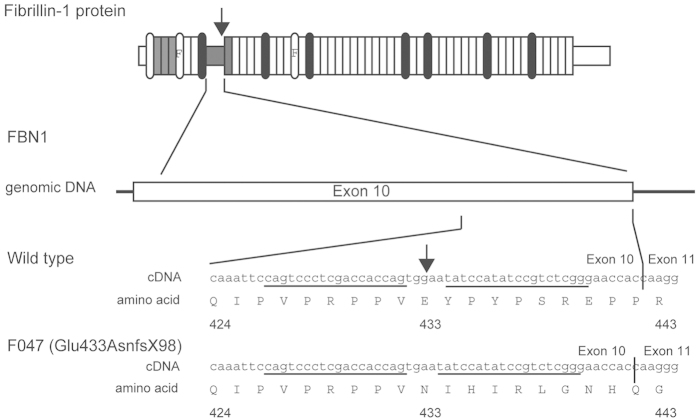
Schematic representation of porcine *FBN1* showing the cleavage site for the zinc finger nuclease FBN1ZFN05. Arrows indicate the cleavage sites for the ZFN. The ZFN recognition sequence is underlined.

We used electroporation to introduce an mRNA encoding the zinc finger nuclease (ZFN) FBN1ZFN05 that targets exon 10 of *FBN1,* which encodes a proline-rich region C (Fig. 1). Seven and 13 clones of homozygous and heterozygous mutants, respectively, which were isolated from 480 colonies, did not display detectable changes in cell shape or proliferation compared with the parental cells (Table 1). We selected the heterozygous *FBN1* mutant clone F047 (+/Glu433AsnfsX98), which encodes a putative truncated FBN1 caused by a 1-bp deletion of a guanine nucleotide that creates a stop codon at amino acid residue 531 (Fig. 1, Fig. S1), to serve as the nuclear donor.

### Generation of cloned piglets with a heterozygous mutant of *FBN1*

*FBN1* mutant cloned piglets with syngeneic backgrounds were created from embryos reconstructed via somatic cell nuclear transfer (SCNT) of heterozygous *FBN1* mutant cells (F047).

In the first experiment, we confirmed that 62.0% (245/395) of the SCNT embryos could develop to the blastocyst stage after *in vitro* culture for 5–6 days. The cloned blastocysts obtained were transferred into two recipients (103 embryos/recipient), and both recipients carried their pregnancies through birth, bearing seven live and one dead offspring (Table 2).

In the second experiment, 334 of the SCNT embryos were cultured *in vitro* for 1–2 days, and 272 embryos at the 1- to 2-cell stage were then transferred into two recipient gilts (136 embryos/recipient). Both recipients became pregnant, and they farrowed a total of 10 live and one stillborn piglets (Table 2).

In both experiments, the efficiencies of generating *FBN1* mutant cloned piglets from SCNT embryos (8/206, 3.9% and 11/272, 4.0%) were within the range of those normally achieved by our laboratory (1.4–4.3%)^25^,^26^,^27^. All *FBN1* mutant cloned piglets harboured the same *FBN1* mutation and were the same sex as the F047 nuclear donor cells (Fig. S1). However, the phenotypes of the *FBN1* mutant cloned pigs obtained in the two experiments differed strikingly as described below.

### Phenotypes of heterozygous *FBN1* mutant cloned pigs derived from blastocysts

**Figure 2:**
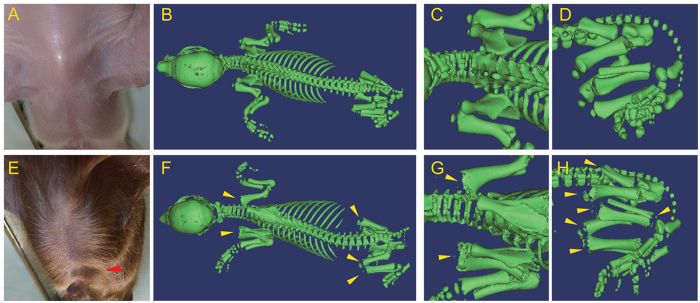
Skeletal abnormalities of the heterozygous *FBN1* mutant cloned pigs. (**A,E**) Appearance of the thorax of a WT pig (**A**) and an *FBN1* mutant pig with pectus excavatum (**E**, red arrowhead). (**B–D,F–H**) Multidetector CT imaging of the skeletal structures of a WT piglet (**B–D**) and an *FBN1* mutant piglet (#6, **F–H**). The WT and *FBN1* mutant piglets had a barrel-shaped (**B**) and a conically shaped (**G**) thorax, respectively. Delayed mineralization of the primary spongiosa and epiphyseal nucleus (arrowhead) was recognized in the humerus (**G**), the os coxa (**H**), the femur (**H**), and the fibula (**H**) of the *FBN1* mutant piglet.

Phenotypic abnormalities in the *FBN1* mutant cloned pigs obtained after transfer of the cloned blastocysts are presented in Table 3. Of the 8 neonatal piglets (including 1 stillborn), 3 exhibited external abnormalities including pectus excavatum (Fig. 2E) or cleft palate (Fig. S2). One of these animals showed both abnormalities.

Four piglets were analysed for skeletal abnormalities by computed tomography (CT) within 3 days after birth. Delayed mineralization in the epiphysis of the os coxa, femur, tibia, fibula, and humerus (Fig. 2G,H) was observed in one animal. Furthermore, the thoraxes of the mutant piglets were nearly conical, whereas the thoraxes of the WT piglets were barrel-shaped. No abnormalities of the spine were observed (Fig. 2B,F).

**Figure 3:**
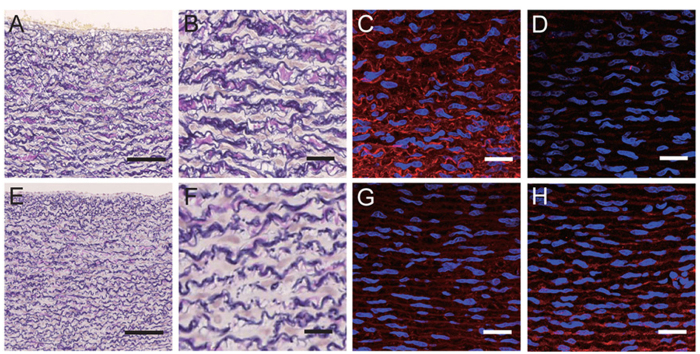
Histology of the proximal thoracic aortic wall of the heterozygous *FBN1* mutant cloned piglets. Proximal thoracic aortic wall of WT (**A–D**) and heterozygous *FBN1* mutant cloned piglets (**E–H**). EVG-stained sections from the *FBN1* mutant cloned piglets (**E,F**) show that the elastic fibres had a fractured and discontinuous structures compared with those of wild-type (WT) piglets (**A,B**). (**C,G**) The intensity of staining of FBN1-positive microfibrils was reduced in the aortic wall medial tissue of the mutant piglets (**G**) compared with that of WT piglets (**C**). (**D,H**) FBN2 was undetectable in the aortic wall medial tissue of the WT piglets (**D**), whereas it was clearly detected in the same tissue of *FBN1* mutant piglets (**H**). Nuclear staining (DAPI) is shown in blue (**C,D,G,H**). Scale bars: 100 μm (**A,E**) and 20 μm (**B–D,F–H**).

Aortic abnormalities were also detected in some of the *FBN1* mutant animals following Elastica van Gieson (EVG) staining of the wall tissue of the proximal thoracic aorta (Fig. 3). The aortic media of the control WT pigs contained a large number of wavy elastic fibres arranged vertically in an orderly structure (Fig. 3A,B). Extensive collagenous connective tissue that stained red was detected between these elastic fibres (Fig. 3A,B). In contrast, the elastic fibres of the aortic medial tissues of the *FBN1* mutant piglets (Fig. 3E,F) exhibited fragmented, discontinuous structures. Furthermore, there was only a sparse collagenous connective tissue layer within the elastic fibre structure (Fig. 3E,F). Immunohistochemical analyses using anti-FBN1 and anti-FBN2 antibodies revealed that the intensity of FBN1-positive microfibrils was lower in the aortic wall medial tissue of the *FBN1* mutant cloned piglets (Fig. 3G) compared with those from the WT piglets (Fig. 3C). In contrast, FBN2 was detected in the tissues of the *FBN1* mutant cloned piglets (Fig. 3H), although it was undetectable in those of the WT controls (Fig. 3D). These histological abnormalities were not detected in the distal thoracic aorta.

Dilation of the sinus of Valsalva and proximal thoracic aorta, as observed in patients with MFS, was not observed in these pigs. No individuals were found to have developed the ectopia lentis generally observed in patients with MFS. Two of the 7 live cloned offspring grew normally beyond sexual maturity.

### Phenotypes of heterozygous *FBN1* mutant cloned pigs derived from early-cleavage stage embryos

No external abnormalities were apparent in any of the 11 *FBN1* mutant neonates (including 1 stillborn) obtained after transfer of the cloned embryos at the early cleavage stages (Table 3). CT analysis of 5 piglets within 4 days after birth detected no skeletal abnormalities.

Three of the cloned pigs exhibited symptoms of gait disturbance, nystagmus, distressed breathing, and quadriplegia and were therefore euthanized at ages ranging from 66 to 90 days. The cause of these abnormalities was unknown.

**Figure 4:**
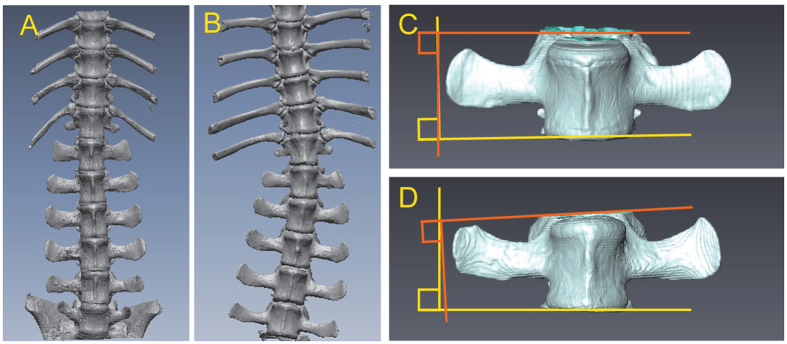
Scoliosis of a *FBN1* mutant cloned pig. (**A,B**) CT image of an *FBN1* mutant cloned pig (#15, **B**) showing scoliosis of the spine, compared with a WT pig (**A**). (**C,D**) CT images of the lumbar vertebrae (L2) of an *FBN1* mutant cloned pig with scoliosis (**D**) indicated that the normal vector of the plane formed by the superior margin of the vertebra and that of the plane formed by the inferior margin were offset by 6.5°. Normal alignment in a WT pig (**C**) was 1.5°.

Two pigs grew to beyond sexual maturity. One was found to exhibit a spinal deformity (Fig. 4B) and abnormal gait at 145 days of age. This pig exhibited astasia at 399 days of age and was therefore euthanized. Scoliosis of the spine was recognized at autopsy, and a CT scan of the spinal column revealed that the normal vector of the plane formed by the superior margin and that of the plane formed by the inferior margin of the lumbar vertebra (second lumber: L2) were offset by 6.5° (normal alignment in pigs, 1.5°) as shown in Fig. 4C,D. The other pig was euthanized at 520 days of age when it exhibited astasia. Pectus excavatum was found at autopsy, although it was not apparent at the neonatal stage.

### Generation of heterozygous and homozygous *FBN1* mutant progeny

We mated heterozygous *FBN1* mutant cloned pigs (founder males) and WT females to produce male and female heterozygous *FBN1* mutant offspring in the first-generation (G1) progeny (Fig. S3). Additionally, the G2 progeny were generated by mating the G1 animals (Fig. S1D, S3, S7A). The G2 offspring comprised homozygous *FBN1* mutants, heterozygous *FBN1* mutants, and wild-type individuals, per Mendelian inheritance principles. The incidence (3 of 12, Table S1) of the heterozygous *FBN1* mutant pigs exhibiting the MFS-like symptoms in the G1 and G2 progeny was similar to that in the founder cloned pigs generated from the short-cultured SCNT embryos (2 of 11). The G2 homozygous mutant pigs showed typical symptoms of MFS, such as dilatation of the ascending aorta and rupture of the elastic lamina (Fig. S4), aortic dissection (Fig. S5), ectopia lentis (Fig. S6), and lipodystrophy (Fig. S7). Similar to the MFS mouse model^10^,^11^,^12^, the homozygous mutation conferred neonatal lethality (longest survival time: 28 days). Furthermore, by examining the levels of *FBN1* mRNA expressed in fibroblasts isolated from these animals, we confirmed that the mRNA had been degraded, probably through a nonsense-mediated mRNA decay (NMD) mechanism (Fig. S8).

## Discussion

There are more than 3,000 documented mutations of human *FBN1* (http://www.umd.be/FBN1/). Patients with MFS with a heterozygous *FBN1* mutation that generates a truncated form of FBN1 (Arg429X, Tyr434X) exhibit typical MFS pathology of the skeleton, eyes, and blood vessels as well as symptoms of scoliosis and dilation of the arteries^28^,^29^. In the present study, to develop an animal model to better understand the pathogenesis of MFS, we generated cloned pigs heterozygous for a mutant *FBN1* (Glu433AsnfsX98).

In the present study, heterozygous *FBN1* mutant cloned pigs were generated from a single line of nuclear donor cells; therefore, all cloned animals were siblings with the same genetic background and the same mutation. However, the cloned piglets did not exhibit a homogenous phenotype. The relationship between MFS genotype and the phenotype is extremely complex, and there are differences in pathology between patients of the same family lineage^30^. Moreover, heterozygous *FBN1* knockout (KO) mice carrying the same mutation exhibit lineage-specific phenotypic differences, and the severity of disease correlates negatively with the level of *FBN1* mRNA expression. These findings suggest the contribution of both epigenetic and genetic factors to the expression of MFS phenotypes and the severity of symptoms^13^.

Immediately following fertilization, paternal genomic DNA undergoes rapid and progressive demethylation, whereas the maternal genomic DNA undergoes gradual demethylation after postfertilization S-phases^31^,^32^. Demethylation progresses until the late morula stage (day 4) followed by remethylation of genomic DNA^31^,^32^. Evidence indicates that the characteristic genomic demethylation–methylation pattern that occurs during early development is affected by *in vitro* culture of embryos. For example, pig embryos cultured *in vitro* and embryos that develop *in utero* differ in their expression of 588 genes^33^. In the present study, we generated cloned pigs from embryos cultured for 1–2 or 5–6 days. Cloned animals derived from the latter cultures more frequently exhibited MFS-like phenotypes. Epigenetic regulatory patterns of transcription are determined early during embryogenesis^31^. Together, these findings suggest that epigenetic factors might have influenced the phenotypes of the *FBN1* mutant cloned pigs generated here. Therefore, we suggest that these differences in phenotypes can be attributed to lower levels of *FBN1* mRNA synthesized by the single WT allele and that efficient generation of porcine models of MFS may require regulation of epigenetic modifiers in addition to mutation of the causal gene. The transcriptional activity of the WT allele in heterozygous *FBN1* mutant pigs needs to be analysed.

To correctly assess the effects of heterozygous *FBN1* mutation in pigs, founder cloned pigs were crossbred with WT pigs to obtain progeny animals, followed by analysis of the offspring’s phenotypes. Because methylation of genomic DNA is normalized by sexual reproduction, this crossbreeding must have eliminated the unnatural epigenetic regulation that resulted from SCNT^31^,^32^. Analysis of the progeny animals therefore enabled us to confirm the influence of the mutant gene in isolation. The heterozygous *FBN1* mutation transmitted from the founder cloned pigs to their progeny caused the similar pathological phenotypes, thereby indicating the significance of this mutation in the pig.

In contrast, dominant incidence of the abnormal phenotype in the founder cloned animals generated from the long-cultured SCNT embryos compared to the progeny animals and the clones derived from the short-cultured embryos further indicated the epigenetic influence on the outcome of the heterozygous *FBN1* mutation.

Scoliosis is a major symptom of MFS and affects more than 60% of patients, and 25–50% of these patients suffer from severe deformities that require surgery^34^. However, the incidence of scoliosis in *FBN1* mutant pigs studied here was lower than that in patients with MFS, and the spinal deformation was less severe. For example, studies of animal models of idiopathic scoliosis indicate that the lower incidence of scoliosis in quadruped animals results from the different directional gravitational force on the spine^35^ between bipedal humans and quadrupedal pigs. Therefore, this difference likely resulted in the lower incidence and reduced severity of scoliosis in the *FBN1* mutant pigs.

Delayed mineralization of the epiphysis occurred in the *FBN1* mutant pigs studied here, which is consistent with studies of mice showing that FBN1 expression levels influence bone density. For example, bone volume is reduced in *Fbn1*^*MgR*/*MgR*^ mice, which express only 15% of the normal level of FBN1 because of TGF-β-induced osteoclastogenesis^36^. Furthermore, bone mineral density and volume are lower in *FBN1*-KO mice than in normal mice^14^, and patients with MFS display reduced mineral density of the epiphysis; however, whether or not this contributes to pathogenesis is unknown^37^. Therefore, a detailed analysis of the bone formation abnormalities that occur in *FBN1* mutant pigs will likely help explain the symptomatology of patients with MFS.

Histopathology of the thoracic aorta is an important characteristic of MFS. For example, dilation of the aorta occurs in 78% of patients with MFS^38^, and enlargement of the aorta and formation of aneurisms may lead to aortic rupture. In patients with MFS with aortic aneurisms, fragmentation of the elastic fibres was evident^39^. Here we observed fragmentation of elastic fibres in the *FBN1* mutant pigs.

Elastic fibres are composed of an elastin core surrounded by microfibrils^40^. FBN2 is present near the centre of the microfibrils, surrounded by FBN1^41^,^42^. Consequently, when centrally located FBN2 epitopes appear exterior to the microfibrils, reaction with anti-FBN2 antibodies is induced. We show here using immunohistochemistry that the tunica media of the thoracic aorta of heterozygous *FBN1* mutant cloned pigs expressed low levels of FBN1 but high levels of FBN2. These findings may be explained as follows: (1) the amount of FBN1 decreased because the *FBN1* mRNA had been degraded, and (2) FBN2 molecules near the central parts of the microfibrils were not covered by a sufficient amount of normal FBN1.

In the present study, we generated pigs that were heterozygous for a mutant *FBN1* and exhibited phenotypes resembling the symptoms of human MFS. Furthermore, two groups of cloned siblings with mutated *FBN1* on a syngeneic background showed striking differences in their individual phenotypes, which is consistent with the distinct phenotypes of familial cases of patients with MFS. The manner in which the expression of FBN1 from the remaining allele is regulated is likely critical for establishing the heterozygous *FBN1* mutant pig as a model for human MFS. Consequently, the heterozygous *FBN1* mutant pigs that we generated in the present study will likely prove useful for investigation of the epigenetic modifiers of MFS onset.

## Material and Methods

### Animal care

The Institutional Animal Care and Use Committee of Meiji University approved the animal experiments described in this study (IACUC12-0008). All animal care and experimental procedures were performed in accordance with Japan Act on Welfare and Management of Animals and regulations. Six crossbred (Large White/Landrace × Duroc) prepubertal gilts served as surrogate mothers for the embryo transfer experiments or as breeder pigs for progeny production. Three male crossbred new-born piglets were used as controls for the *FBN1* mutant cloned piglets. Pigs were housed in a temperature-controlled room, had free access to water and were provided with growth-stage appropriate commercial feed (Chubushiryo Co., Ltd. Nagoya, Japan) in accordance with the Japanese Feeding Standard for Swine (2005)^43^. The health of all pigs was assessed at feeding (08:00 and 17:00). For autopsies, piglets were anaesthetized using intramuscular injection of ketamine (11 mg/kg, Fujita Pharmaceutical Co., Ltd., Tokyo, Japan). Anaesthesia was maintained via inhalation of isoflurane (DS Pharma animal Health Co., Ltd., Osaka, Japan), and the piglets were exsanguinated. Adult pigs were administered intramuscular injection of 1% mafoprazine mesylate (0.5 mg/kg, DS Pharma animal Health Co., Ltd.) followed by intravenous pentobarbital (Kyoritsu Seiyaku Co., Ltd., Tokyo, Japan), and anaesthesia was maintained via inhalation of isoflurane while the pigs were exsanguinated.

### Chemicals

All chemicals were purchased from the Sigma-Aldrich Chemical Co. (St. Louis, MO, USA) unless otherwise indicated.

### Design **of ZFNs**

A custom ZFN mRNA for porcine *FBN1* was obtained from Sigma-Aldrich Chemical Co. The supplier designed and validated the ZFN mRNAs. Each ZFN contained six zinc finger domains recognizing 18 bases (Fig. 1). These sequences displayed high specificity, such that if fewer than 9 of the 36 bases were mismatched, off-target sequences were not recognized. This specificity was confirmed using the porcine genome DNA database Sscrofa9 Ensembl (http://feb2012.archive.ensembl.org/Sus_scrofa/Info/Index) (Table S2).

### Isolation **of** ***FBN1*** **mutant cells and culture conditions**

A primary culture of porcine foetal fibroblasts (male, Large White/Landrace × Duroc) was used to isolate *FBN1* mutant cells. The fibroblasts and their derivatives (mutant cells) were seeded on type I collagen-coated dishes or plates (Asahi Glass, Tokyo, Japan) and were cultured in MEMα (Life Technologies, Carlsbad, CA, USA) supplemented with 15% foetal bovine serum (FBS, Nichirei Bioscience, Tokyo, Japan) and 1× antibiotic-antimycotic solution (Life Technologies) in a humidified atmosphere containing 5% CO_2_ at 37 °C. The foetal fibroblasts were cultured to 70–90% confluence, washed twice with D-PBS(−) (Life Technologies) and treated with 0.05% trypsin-EDTA (Life Technologies) to isolate and collect the cells. The cells (4 × 10^5^) were then suspended in 40 μL of R buffer (a component of the Neon Transfection System, Life Technologies), and 2 μL of ZFN-encoding mRNA solution (400 ng/μL in RNase-free water) was added. The cells were electroporated as follows: pulse voltage, 1,100 V; pulse width, 30 ms; and pulse number, 1. Following electroporation, the cells were cultured at 32 °C for 3 days (transient cold shock), initially without antibiotics in the medium described above and then with antibiotics after 24 h^44^. For recovery from the transient cold shock, the cells were cultured at 37 °C until they approached confluence. Five 96-well plates were used to prepare a limiting dilution of a cell suspension, and 12 days later, cells that were >50% confluent in each well were selected and subcultured.

### Analysis of ZFN-induced mutations in nuclear donor cells and cloned piglets

The target region of *FBN1*-ZFNs was amplified via direct PCR from the cell clones using MightyAmp DNA polymerase (Takara Bio, Shiga, Japan) and the corresponding primers 5′-GACATAGGTGAAGACTTCGTAGG and 5′-TCACTCTCAAGACTCCAGTTTGG. Nested PCR was performed using PrimeSTAR HS DNA polymerase (Takara Bio) and the appropriate primers 5′-AACTGAGAGTGACTTCCATGGAC and 5′-GCAACCGCTCATTTTCCTCTATG. The nested PCR fragment from each clone was analysed using the sequencing primer 5′-TAACTTGTGCTCCAGCTCAGGTG, the BigDye Terminator Cycle Sequencing Kit and an ABI PRISM 3130xl Genetic Analyzer (Life Technologies). The nested PCR fragments that contained the mutation were subcloned into the sequencing vector pCR4Blunt-TOPO (Life Technologies).

For analysis of the mutation in each piglet, genomic DNA was extracted from tail biopsies of the piglets using a DNeasy Tissue and Blood Kit (QIAGEN, Hilden, Germany), and PCR genotyping and DNA sequencing were performed as described above.

### Somatic cell nuclear transfer and embryo transfer

SCNT was performed as described previously with slight modifications^26^. Briefly, *in vitro*-matured oocytes were enucleated via gentle aspiration of the polar body and the adjacent cytoplasm using a bevelled pipette in Tyrode lactose medium containing 10 mM HEPES and 0.3% (w/v) polyvinylpyrrolidone in the presence of 0.1 μg/mL demecolcine, 5 μg/mL cytochalasin B (CB) and 10% FBS.

Nuclear donor cells (clone F047) were used following cell-cycle synchronization induced by serum starvation for two days. A single donor cell was inserted into the perivitelline space of an enucleated oocyte. The donor cell-oocyte complexes were placed in a solution containing 280 mM mannitol (Nacalai Tesque, Kyoto, Japan), 0.15 mM MgSO_4_, 0.01% (w/v) polyvinyl alcohol (PVA) and 0.5 mM HEPES (pH 7.2) and were held between two electrode needles. Membrane fusion was induced using an Electro Cell Fusion Generator (LF201; Nepa Gene, Chiba, Japan) by applying a single pulse of direct-current (DC) (267 V/mm for 20 μs) and pre- and post-pulse alternating currents of 4 V at 1 MHz for 5 s.

The reconstructed embryos were cultured in porcine zygote medium-5 (PZM-5; Research Institute for the Functional Peptides, Yamagata, Japan) supplemented with 4 mg/mL bovine serum albumin for 1 to 1.5 h, followed by electrical activation. For induction of electrical activation, the reconstructed embryos were aligned between two wire electrodes (1.0 mm apart) on a fusion chamber slide filled with the activation solution (280 mM mannitol, 0.05 mM CaCl_2_, 0.1 mM MgSO_4_ and 0.01% (w/v) PVA). A DC pulse of 150 V/mm was applied for 100 μs using the Electro Cell Fusion Generator. After activation, the reconstructed embryos were cultured in PZM-5 for 3 h in 5 μg/mL CB and 500 nM Scriptaid followed by culture in 500 nM Scriptaid for 12–14 h. To assess their development, the cloned embryos were next cultured in PZM-5 and divided into two groups (SCNT embryos cultured for 1–2 days or 5–6 days). Embryo culture was performed in a humidified atmosphere containing 5% CO_2_, 5% O_2_, and 90% N_2_ at 38.5 °C. After reaching the morula stage, the embryos were cultured in PZM-5 supplemented with 10% FBS.

Crossbred prepubertal gilts weighing 100 to 105 kg were used as recipients of the SCNT embryos after synchronization of oestrus by administration of equine chorionic gonadotropin (eCG, ASKA Pharmaceutical, Tokyo, Japan) and human chorionic gonadotropin (hCG, Kyoritsu Pharmaceutical, Tokyo, Japan). The SCNT embryos at the 1- to 2-cell stage were transferred to the oviduct of the recipients after culture for 1–2 days. After culture for 5–6 days, the SCNT blastocysts were transferred to the uterine horns of the recipients. Embryos were transferred to pigs administered general anaesthesia induced using intramuscular injection of mafoprazine mesylate and thiopental (10 mg/kg, DS Pharma animal Health Co., Ltd., Osaka, Japan), and anaesthesia was maintained by administration of isoflurane gas.

### CT analysis

CT analyses of the piglets were performed using a SOMATOM Sensation 16 CT scanner (Siemens AG, Munich, Germany). Piglets were subjected to CT after death. Each specimen was placed in a prone position, and noncontrast CT scans of sections 0.625-mm thick were obtained from vertical axial tomographs acquired at 0.625-mm intervals. Scan settings were 120 kVp, 400 mA, and a tube rotation time of 0.5 s. The grey-scale threshold was set to 75 to separate bone from the surrounding tissue using an adaptive threshold method. Three-dimensional (3D) models of the whole body were based on the 0.625-mm thick CT scan slices generated using Advantage Workstation 4.5 software (GE Healthcare, Waukesha, WI, USA).

### Analysis of scoliosis

The spines of pigs potentially suffering from scoliosis were removed during autopsy and analysed using CT. DICOM (Digital Imaging and Communication in Medicine) data were converted to Stereolithography data using AVIZO (FEI Company, Hillsboro, OR, USA). RapidFormORX (Geomagic Inc., Morrisville, NC, USA) was used to determine the 3D coordinates of the upper and lower surfaces of each vertebral body, and these data were used to derive equations for the planes of best fit for each vertebral body. The inner product of the normal unit vectors of the upper and lower planes was calculated, the angle formed by the two planes was determined and the deformity of the lumbar vertebrae was assessed.

### Histological analysis

Thoracic aortic tissues were fixed in 10%-neutral buffered formalin solution (Wako Pure Chemical Industries, Osaka, Japan), embedded in paraffin, sectioned, and treated with EVG stain using standard methods. The primary antibodies used were an anti-FBN1 polyclonal antibody (provided by Prof. Tomoyuki Nakamura, Kansai Medical University)^45^ and an anti-FBN2 polyclonal antibody (provided by Dr. Lynn Sakai, Shriner Institute)^42^. The secondary antibody used was an Alexa Fluor 555-conjugated anti-rabbit IgG (Life Technologies), and the nuclei were stained with 4′,6-diamidino-2-phenylindole (DAPI). Images were acquired using a confocal microscope with 488- and 543-nm filters (LSM780; Carl Zeiss MicroImaging, Jena, Germany).

## Additional Information

**How to cite this article**: Umeyama, K. *et al.* Generation of heterozygous fibrillin-1 mutant cloned pigs from genome-edited foetal fibroblasts. *Sci. Rep.* **6**, 24413; doi: 10.1038/srep24413 (2016).

## Supplementary Material

Supplementary Information

## Figures and Tables

**Table 1:** Sequencing assay for ZFN-induced mutations in the FBN1-targeted region.

Wild type genomic DNA		
	CAGTCCCTCGACCACCAGTGGAATATCCATATCCGTCTCGG	
FBN1ZFN05 binding sequence		
	CAGTCCCTCGACCACCAGnnnnnTATCCATATCCGTCTCGG	
Homozygous mutants		
F038	CAGTCCCTCGACCACCAG*****TATCCATATCCGTCTCGG	5bp del
F063	CAGTCCCTCGACCA***********TCCATATCCGTCTCGG	11bp del
F162	CAGTCCCTCGACCA***************TATCCGTCTCGG	15bp del
F007	CAGTCCCTCGACCACCAGACGGATATCCATATCCGTCTCGG	4bp sub
	CAGTCCCTCGACCAC*************ATATCCGTCTCGG	13bp del
F022	CAGTCCCTCGACCACCAG****ATATCCATATCCGTCTCGG	4bp del
	CAGTCCCTCGACC*****TGGAATATCCATATCCGTCTCGG	5bp del
F083	CAGTCCCTCGACCAC****GGAATATCCATATCCGTCTCGG	4bp del
	CAGTCCCTCGACC**************CATATCCGTCTCGG	14bp del
F117	CAGTCCCTCGACCACCA************TATCCGTCTCGG	12bp del
	CAGTCCCTCGACCACCAG*************TCCGTCTCGG	13bp del
Heterozygous mutants		
F002	CAGTCCCTCGACCACCA***(111bp del)**TTAAGTCA	111bp del
F005	CAGTCCCTCGACCACCAGTGGATGGAATATCCATATCCGTCTCGG	4bp ins
F013	CAGTCCCTCGACCACCAG***AATATCCATATCCGTCTCGG	3bp del
F020	CAGTCCCTCGACCACCAGT*****ATCCATATCCGTCTCGG	5bp del
F029	CAGTCCCTCGACCA**(63del + 7bp ins)**CTTTTC	56bp del
F035	CAGTCCCTCGACCACCAGTG***TATCCATATCCGTCTCGG	3bp del
F036	CAGTCCCTCGACCACCAGTGGAATGGAATATCCATATCCGTCTCGG	5bp ins
F041	CAGTCCCTCGACCACCAG****ATATCCATATCCGTCTCGG	4bp del
F043	CAGTCCCTCGACCACCA******TATCCATATCCGTCTCGG	6bp del
F047	CAGTCCCTCGACCACCAGTG*AATATCCATATCCGTCTCGG	1bp del
F050	CAGTCCCTCGACCACCAG**GAATATCCATATCCGTCTCGG	2bp del
F074	CAGTCCCTCGACCACCAGTGGAA*ATCCATATCCGTCTCGG	1bp del
F098	CAGTCCCTCGACCACCAG****ATATCCATATCCGTCTCGG	4bp del

Multiple deletions or insertions depicted using asterisks or underlines, respectively.

**Table 2:** *In vitro* development of SCNT embryos and production of heterozygous *FBN1* mutant cloned piglets.

*In vitro* development of SCNT embryos				
SCNT embryos reconstructed	395		334	
Early cleavage-stage embryos	302		272^b^	
Blastocyst-stage embryos on day 5	245^a^		–	
Production of heterozygous *FBN1* mutant cloned piglets				
Recipient	#R1^a^	#R2^a^	#R3^b^	#R4^b^
Embryos transferred	103	103	136	136
Pregnancy	+	+	+	+
No. of viable cloned piglet	4	3	2	8
No. of stillborn cloned piglet	0	1	1	0

^a^The SCNT embryos cultured for 5 to 6 days were transferred to the recipients’ uteri.

^b^The SCNT embryos cultured for 1 to 2 days were transferred to the recipients’ oviducts.

**Table 3:** Phenotypes of heterozygous *FBN1* mutant cloned piglets.

Piglet No.	Birth weight (kg)	Postpartum survival (day)	Neonatal phenotypic abnormalities	Phenotypic features
Embryo transfer at 5 or 6 days				
#1	1.088	551^e^	−	Quadriplegia
#2	1.051	19^u^	+	Fragmentation of elastic fibers*, Pectus excavatum*
#3	0.550	68^u^	−	
#4	0.536	1^e^	+	Cleft palate, Fragmentation of elastic fibers*, Pectus excavatum*
#5	0.858	738^e^	−	
#6	0.911	3^u^	+	Delayed bone mineralization*
#7	0.944	0^s^	+	Cleft palate
#8	0.811	0^u^	−	
Embryo transfer at 1 or 2 days				
#9	1.481	90^e^	−	Quadriplegia
#10	1.247	520^e^	−	Pectus excavatum*, Quadriplegia
#11	0.506	0^s^	−	
#12	0.855	4^u^	−	
#13	0.956	76^e^	−	Distressed breathing, Quadriplegia
#14	0.605	0^u^	−	
#15	0.875	412^e^	−	Scoliosis*
#16	1.210	3^u^	−	
#17	1.158	0^e^	−	
#18	0.781	38^u^	−	
#19	0.849	66^e^	−	Nystagmus, Quadriplegia

^e^Euthanatized.

^s^Stillborn.

^u^The cause of death is unknown.

^*^Marfan-like symptoms.

## References

[b1] Sakai L. Y., Keene D. R. & Engvall E. Fibrillin, a new 350-kD glycoprotein, is a component of extracellular microfibrils. J Cell Biol 103, 2499–2509 (1986). 3536967 10.1083/jcb.103.6.2499 PMC2114568

[b2] Robinson P. N. & Booms P. The molecular pathogenesis of the Marfan syndrome. Cell Mol Life Sci 58, 1698–1707 (2001). 11706995 10.1007/PL00000807 PMC11337295

[b3] Ramirez F. & Dietz H. C. Fibrillin-rich microfibrils: Structural determinants of morphogenetic and homeostatic events. J Cell Physiol 213, 326–330 (2007). 17708531 10.1002/jcp.21189

[b4] Neptune E. R. *et al.* Dysregulation of TGF-beta activation contributes to pathogenesis in Marfan syndrome. Nat Genet 33, 407–411 (2003). 12598898 10.1038/ng1116

[b5] Kaartinen V. & Warburton D. Fibrillin controls TGF-beta activation. Nat Genet 33, 331–332 (2003). 12610545 10.1038/ng0303-331

[b6] Dietz H. C. *et al.* Marfan syndrome caused by a recurrent de novo missense mutation in the fibrillin gene. Nature 352, 337–339 (1991). 1852208 10.1038/352337a0

[b7] Lee B. *et al.* Linkage of Marfan syndrome and a phenotypically related disorder to two different fibrillin genes. Nature 352, 330–334 (1991). 1852206 10.1038/352330a0

[b8] Dean J. C. Marfan syndrome: clinical diagnosis and management. Eur J Hum Genet 15, 724–733 (2007). 17487218 10.1038/sj.ejhg.5201851

[b9] Canadas V., Vilacosta I., Bruna I. & Fuster V. Marfan syndrome. Part 1: pathophysiology and diagnosis. Nat Rev Cardiol 7, 256–265 (2010). 20351703 10.1038/nrcardio.2010.30

[b10] Pereira L. *et al.* Targetting of the gene encoding fibrillin-1 recapitulates the vascular aspect of Marfan syndrome. Nat Genet 17, 218–222 (1997). 9326947 10.1038/ng1097-218

[b11] Judge D. P. *et al.* Evidence for a critical contribution of haploinsufficiency in the complex pathogenesis of Marfan syndrome. J Clin Invest 114, 172–181 (2004). 15254584 10.1172/JCI20641 PMC449744

[b12] Carta L. *et al.* Fibrillins 1 and 2 perform partially overlapping functions during aortic development. J Biol Chem 281, 8016–8023 (2006). 16407178 10.1074/jbc.M511599200 PMC3052983

[b13] Lima B. L. *et al.* A new mouse model for marfan syndrome presents phenotypic variability associated with the genetic background and overall levels of Fbn1 expression. PLoS One 5, e14136 (2010). 21152435 10.1371/journal.pone.0014136 PMC2994728

[b14] Cook J. R. *et al.* Generation of Fbn1 conditional null mice implicates the extracellular microfibrils in osteoprogenitor recruitment. Genesis 50, 635–641 (2012). 22374917 10.1002/dvg.22022 PMC3405165

[b15] Yang D. *et al.* Generation of PPARgamma mono-allelic knockout pigs via zinc-finger nucleases and nuclear transfer cloning. Cell Res 21, 979–982 (2011). 21502977 10.1038/cr.2011.70 PMC3203707

[b16] Hauschild J. *et al.* Efficient generation of a biallelic knockout in pigs using zinc-finger nucleases. Proc Natl Acad Sci USA 108, 12013–12017 (2011). 21730124 10.1073/pnas.1106422108 PMC3141985

[b17] Lutz A. J. *et al.* Double knockout pigs deficient in N-glycolylneuraminic acid and galactose alpha-1,3-galactose reduce the humoral barrier to xenotransplantation. Xenotransplantation 20, 27–35 (2013). 23384142 10.1111/xen.12019

[b18] Watanabe M. *et al.* Generation of interleukin-2 receptor gamma gene knockout pigs from somatic cells genetically modified by zinc finger nuclease-encoding mRNA. PLos One 8, e76478 (2013). 24130776 10.1371/journal.pone.0076478 PMC3793986

[b19] Carlson D. F. *et al.* Efficient TALEN-mediated gene knockout in livestock. Proc Natl Acad Sci USA 109, 17382–17387 (2012). 23027955 10.1073/pnas.1211446109 PMC3491456

[b20] Lee K. *et al.* Engraftment of human iPS cells and allogeneic porcine cells into pigs with inactivated RAG2 and accompanying severe combined immunodeficiency. Proc Natl Acad Sci USA 111, 7260–7265 (2014). 24799706 10.1073/pnas.1406376111 PMC4034252

[b21] Tan W. *et al.* Efficient nonmeiotic allele introgression in livestock using custom endonucleases. Proc Natl Acad Sci USA 110, 16526–16531 (2013). 24014591 10.1073/pnas.1310478110 PMC3799378

[b22] Whitworth K. M. *et al.* Use of the CRISPR/Cas9 system to produce genetically engineered pigs from *in vitro*-derived oocytes and embryos. Biol Reprod 91, 78 (2014). 25100712 10.1095/biolreprod.114.121723 PMC4435063

[b23] Li P. *et al.* Efficient generation of genetically distinct pigs in a single pregnancy using multiplexed single-guide RNA and carbohydrate selection. Xenotransplantation 22, 20–31 (2015). 25178170 10.1111/xen.12131

[b24] Zhou X. *et al.* Generation of CRISPR/Cas9-mediated gene-targeted pigs via somatic cell nuclear transfer. Cell Mol Life Sci 72, 1175–1184 (2015). 25274063 10.1007/s00018-014-1744-7 PMC11113635

[b25] Kurome M. *et al.* Production efficiency and telomere length of the cloned pigs following serial somatic cell nuclear transfer. J Reprod Dev 54, 254–258 (2008). 18490858 10.1262/jrd.20038

[b26] Matsunari H. *et al.* Transgenic-cloned pigs systemically expressing red fluorescent protein, Kusabira-Orange. Cloning Stem Cells 10, 313–323 (2008). 18729767 10.1089/clo.2008.0024

[b27] Watanabe M. *et al.* Production of transgenic cloned pigs expressing the far-red fluorescent protein monomeric Plum. J Reprod Dev 61, 169–177 (2015). 25739316 10.1262/jrd.2014-153 PMC4498373

[b28] Liu W. O., Oefner P. J., Qian C., Odom R. S. & Francke U. Denaturing HPLC-identified novel FBN1 mutations, polymorphisms, and sequence variants in Marfan syndrome and related connective tissue disorders. Genet Test 1, 237–242 (1997). 10464652 10.1089/gte.1997.1.237

[b29] Rommel K., Karck M., Haverich A., Schmidtke J. & Arslan-Kirchner M. Mutation screening of the fibrillin-1 (FBN1) gene in 76 unrelated patients with Marfan syndrome or Marfanoid features leads to the identification of 11 novel and three previously reported mutations. Hum Mutat 20, 406–407 (2002). 12402346 10.1002/humu.9075

[b30] Potter K. J. *et al.* The c.7409G > A (p.Cys2470Tyr) Variant of FBN1: Phenotypic Variability across Three Generations. Mol Syndromol 4, 125–135 (2013). 23653584 10.1159/000347163 PMC3638931

[b31] Yang X. *et al.* Nuclear reprogramming of cloned embryos and its implications for therapeutic cloning. Nat Genet 39, 295–302 (2007). 17325680 10.1038/ng1973

[b32] Cao Z. *et al.* Dynamic reprogramming of 5-hydroxymethylcytosine during early porcine embryogenesis. Theriogenology 81, 496–508 (2014). 24315686 10.1016/j.theriogenology.2013.10.025

[b33] Bauer B. K. *et al.* Transcriptional profiling by deep sequencing identifies differences in mRNA transcript abundance in *in vivo*-derived versus *in vitro*-cultured porcine blastocyst stage embryos. Biol Reprod 83, 791–798 (2010). 20668257 10.1095/biolreprod.110.085936

[b34] Gjolaj J. P. *et al.* Spinal deformity correction in Marfan syndrome versus adolescent idiopathic scoliosis: learning from the differences. Spine (Phila Pa 1976) 37, 1558–1565 (2012). 22426454 10.1097/BRS.0b013e3182541af3

[b35] Janssen M. M., de Wilde R. F., Kouwenhoven J. W. & Castelein R. M. Experimental animal models in scoliosis research: a review of the literature. Spine J 11, 347–358 (2011). 21474088 10.1016/j.spinee.2011.03.010

[b36] Nistala H. *et al.* Differential effects of alendronate and losartan therapy on osteopenia and aortic aneurysm in mice with severe Marfan syndrome. Hum Mol Genet 19, 4790–4798 (2010). 20871099 10.1093/hmg/ddq409 PMC2989889

[b37] Haine E. *et al.* Muscle and bone impairment in children with Marfan syndrome: correlation with age and FBN1 genotype. J Bone Miner Res 30, 1369–1376 (2015). 25656438 10.1002/jbmr.2471

[b38] Hwa J. *et al.* The natural history of aortic dilatation in Marfan syndrome. Med J Aust 158, 558–562 (1993). 8487722 10.5694/j.1326-5377.1993.tb121876.x

[b39] Bunton T. E. *et al.* Phenotypic alteration of vascular smooth muscle cells precedes elastolysis in a mouse model of Marfan syndrome. Circ Res 88, 37–43 (2001). 11139471 10.1161/01.res.88.1.37

[b40] Wu D., Shen Y. H., Russell L., Coselli J. S. & LeMaire S. A. Molecular mechanisms of thoracic aortic dissection. J Surg Res 184, 907–924 (2013). 23856125 10.1016/j.jss.2013.06.007 PMC3788606

[b41] Charbonneau N. L. *et al.* *In vivo* studies of mutant fibrillin-1 microfibrils. J Biol Chem 285, 24943–24955 (2010). 20529844 10.1074/jbc.M110.130021 PMC2915730

[b42] Charbonneau N. L. *et al.* Microfibril structure masks fibrillin-2 in postnatal tissues. J Biol Chem 285, 20242–20251 (2010). 20404337 10.1074/jbc.M109.087031 PMC2888437

[b43] National Agriculture and Bio-oriented Research Organization. Japanese Feeding Standard for Swine. (Japan Livestock Industry Association, Tokyo) (2005).

[b44] Doyon Y. *et al.* Transient cold shock enhances zinc-finger nuclease-mediated gene disruption. Nat Methods 7, 459–460 (2010). 20436476 10.1038/nmeth.1456

[b45] Inoue T. *et al.* Latent TGF-beta binding protein-2 is essential for the development of ciliary zonule microfibrils. Hum Mol Genet 23, 5672–5682 (2014). 24908666 10.1093/hmg/ddu283 PMC4189902

