# Supplementary material for: Generation of heterozygous fibrillin-1 mutant cloned pigs from genome-edited foetal fibroblasts
Source: Sci Rep. 2016 Apr 14;6:24413. doi: 10.1038/srep24413 (PMC4830947; doi:10.1038/srep24413)
Supplement: Supplementary Information [file srep24413-s1.pdf]

Supplementary information

Generation of heterozygous fibrillin-1 mutant cloned pigs from genome-edited foetal fibroblasts.

Kazuhiro Umeyama[1,2], Kota Watanabe[3], Masahito Watanabe[1,2], Keisuke Horiuchi[3,4], Kazuaki Nakano[2], Masateru Kitashiro[3], Hitomi Matsunari[1,2], Tokuhiro Kimura[5,#a], Yoshimi Arima[6], Oltea Sampetrean[6], Masaki Nagaya[1], Masahiro Saito[7], Hideyuki Saya[6], Kenjiro Kosaki[8], Hiroshi Nagashima[1,2*], Morio Matsumoto[3*]

Supplementary table

Table S1. Phenotypes of *FBN1* mutant progeny.

Heterozygous mutant progeny

Piglet No.	Birth weight (kg)	Sex	Genotype	Postpartum survival (day)	Phenotypic abnormalities	Phenotypic features
G1-3	1.635	M	Wt/Mut	165[e]	-	
G1-5	1.733	M	Wt/Mut	165[e]	-	
G1-6	1.316	F	Wt/Mut	>885	NA	
G1-7	1.789	M	Wt/Mut	165[e]	+	Fragmentation of elastic fibres
G1-8	1.805	M	Wt/Mut	266[e]	-	
G1-10	1.498	F	Wt/Mut	266[e]	-	
G1-12	1.443	F	Wt/Mut	180[u]	+	Scoliosis
G1-13	1.788	M	Wt/Mut	>810	NA	
G1-18	1.025	M	Wt/Mut	1[a]	-	
G2-2	1.428	F	Wt/Mut	28[e]	-	
G2-4	1.647	M	Wt/Mut	28[e]	-	
G2-6	1.262	F	Wt/Mut	28[e]	-	
G2-7	1.280	M	Wt/Mut	28[e]	-	
G2-11	1.448	M	Wt/Mut	0[a]	+	Fragmentation of elastic fibres

Homozygous mutant progeny

Piglet No.	Birth weight (kg)	Sex	Genotype	Postpartum survival (day)	Phenotypic abnormalities	Phenotypic features
G2-3	1.341	M	Mut/Mut	2[a]	+	Fragmentation of elastic fibres
G2-5	1.485	F	Mut/Mut	28[e]	+	Ectopia lentis, Fragmentation of elastic fibres, Lipodystrophy
G2-9	1.469	F	Mut/Mut	0[a]	+	Aortic dissection, Fragmentation of elastic fibres,
G2-10	1.725	M	Mut/Mut	0[s]	+	Fragmentation of elastic fibres, Lipodystrophy

[a] accidental death, [e] euthanazized, [s] stillborn, [u] cause of death unknown.

NA: not analysed.

Table S2. Number of potential off-target sites for the zinc finger nuclease FBN1ZFN05 in the porcine genome.

		No. of mismatches for ZFN heterodimers										
Target code	Right / left	0	1	2	3	4	5	6	7	8	9	10
FBN1ZFN05	aaTATCCATATCCGTCTCGGgaaccacc / caCTGGTGGTCGAGGGACTGgaatttgc	1	0	0	0	0	0	0	0	0	1	12

Supplementary figures

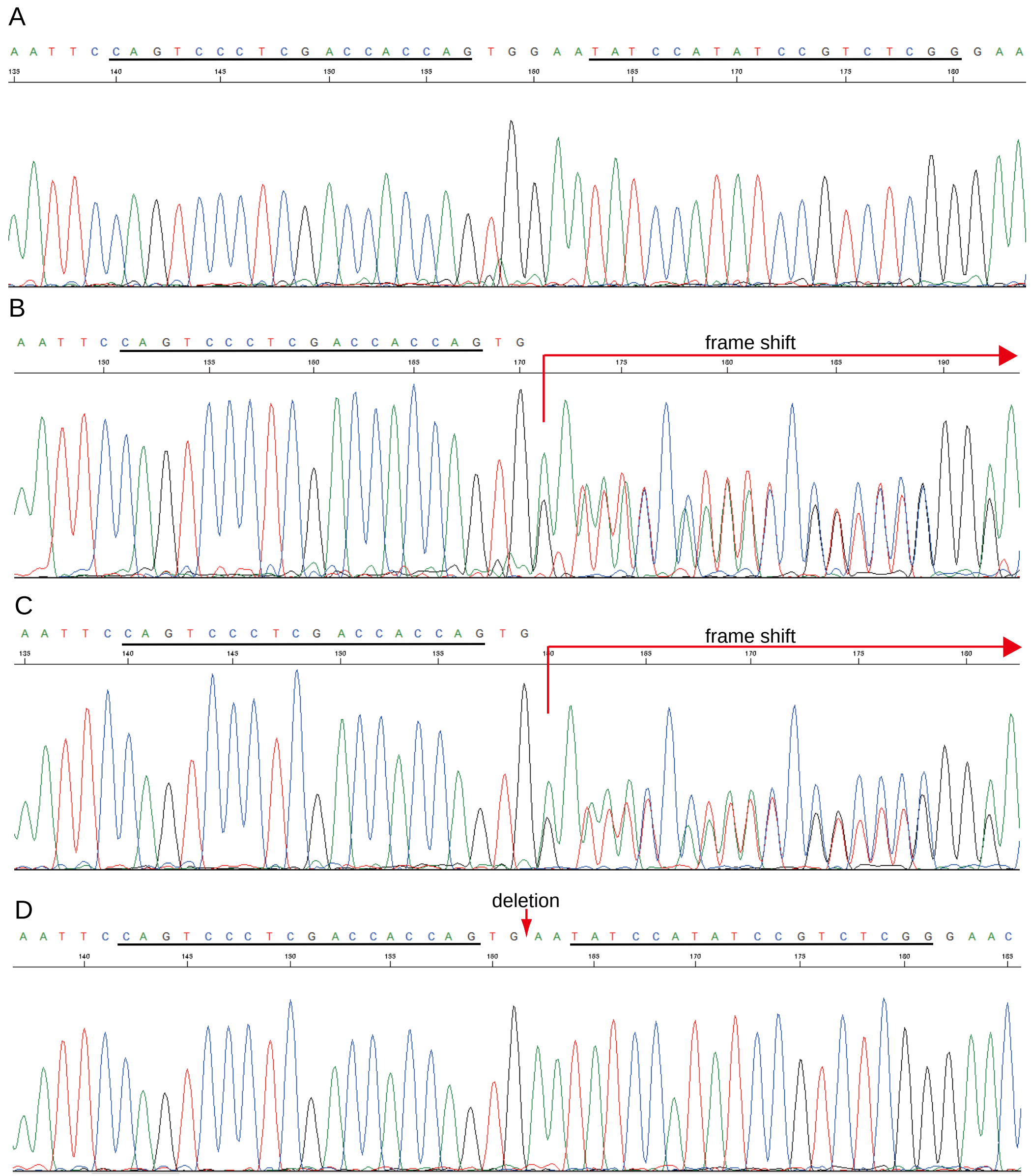

Figure S1. DNA sequence analysis of *FBN1* mutants.

(**A**) Wild-type cells. (**B**) Heterozygous *FBN1* mutant cells (F047). (**C**) Heterozygous *FBN1* mutant cloned piglets. (**D**) Homozygous *FBN1* mutant piglets. The underlined portions indicate the ZFN (FBN1ZFN05)-binding sequence.

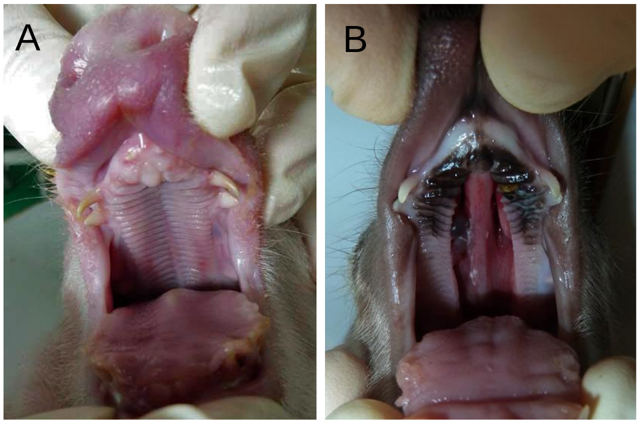

Figure S2. Dysplasia of the hard palate in the *FBN1* mutant cloned piglets.

Hard palates of a WT (**A**) and an *FBN1* mutant cloned piglet (**B**) with the typical features of cleft palate.

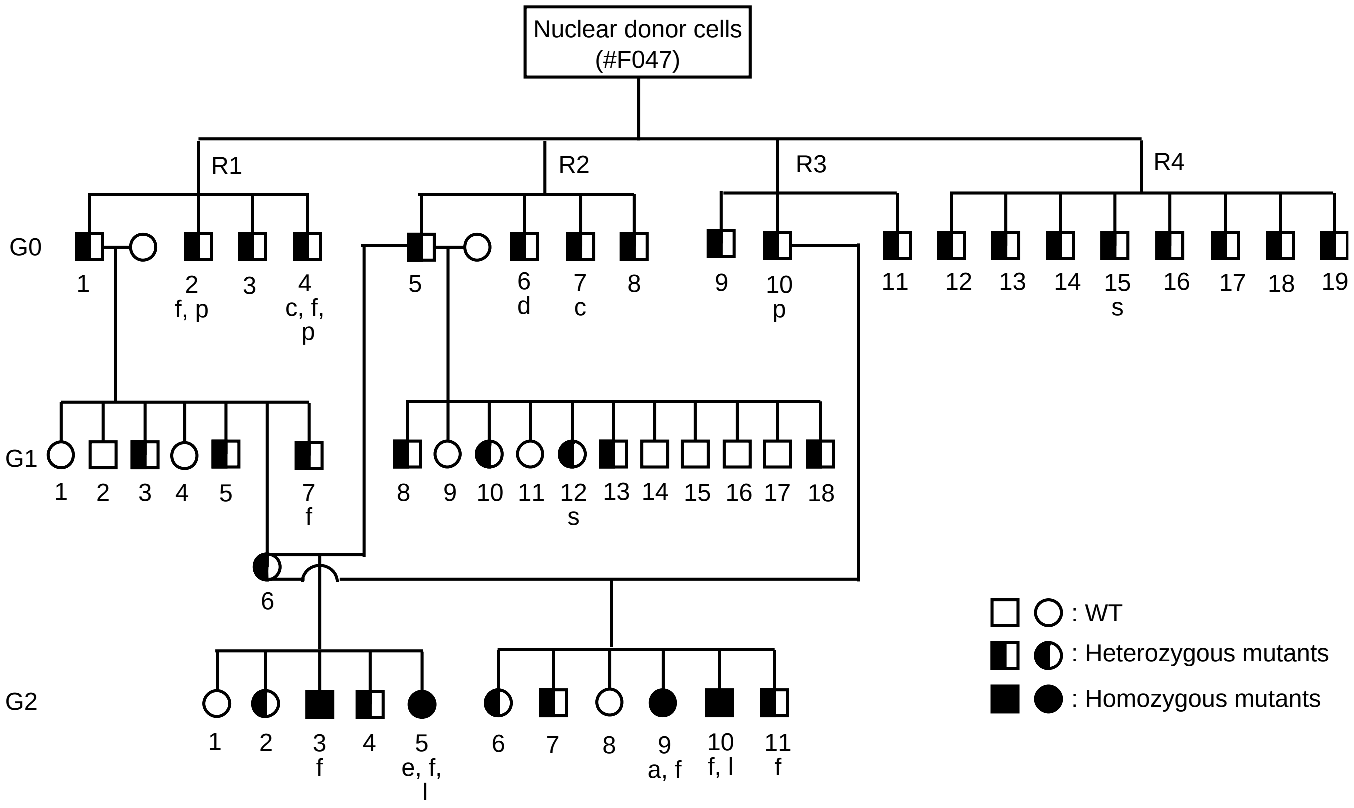

Figure S3. Pedigree chart of *FBN1* mutant pigs.

(R1, R2) *FBN1* mutant cloned pigs generated from SCNT embryos transferred after culture to the blastocyst stage. (R3, R4) *FBN1* mutant cloned pigs generated from SCNT embryos transferred at the early-cleavage stage. Squares and circles indicate males and females, respectively.

a, Aortic dissection; c, cleft palate; d, delayed bone mineralization; e, ectopia lentis; f, fragmentation of elastic fibres; l, lipodystrophy; p, pectus excavatum; and s, scoliosis.

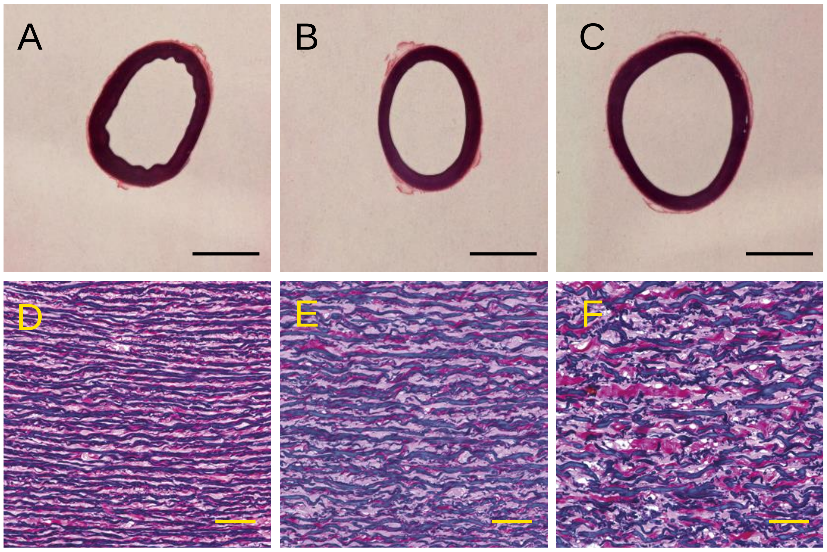

Figure S4. Aortic wall abnormality in the homozygous *FBN1* mutant pig.

(**A**, **B**, **C**) Cross-sectional areas of the ascending aorta in an age-matched WT (**A**, body weight (BW): 8.8 kg), heterozygous mutant (**B**, BW: 7.1 kg), and homozygous mutant pig (**C**, BW: 4.35 kg) were 40.3, 41.0, and 72.8 mm^2 respectively. Respective histology of the wall of the ascending aorta is shown (**D**, **E**, **F**; EVG stain). Fractured and discontinuous structure of aortic medial tissue was noticeable in the homozygous *FBN1* mutant pig (**F**), whereas symptoms were moderate in the heterozygous *FBN1* mutant pigs (**E**). Scale bars: 5 mm (**A**, **B**, **C**) and 40 μm (**D**, **E**, **F**).

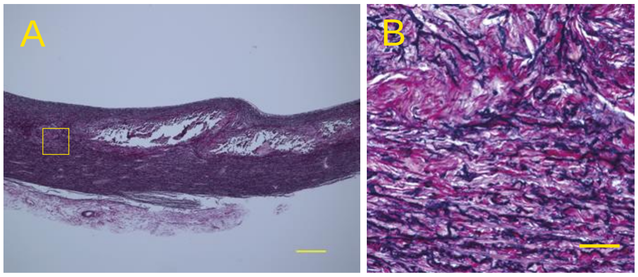

Figure S5. Aortic dissection in a homozygous *FBN1* mutant pig.

(**A**) Histology of the wall of the ascending aorta stained by EVG. (**B**) Magnified view of the aortic medial tissue outlined in A. Scale bars: 300 μm (**A**) and 40 μm (**B**).

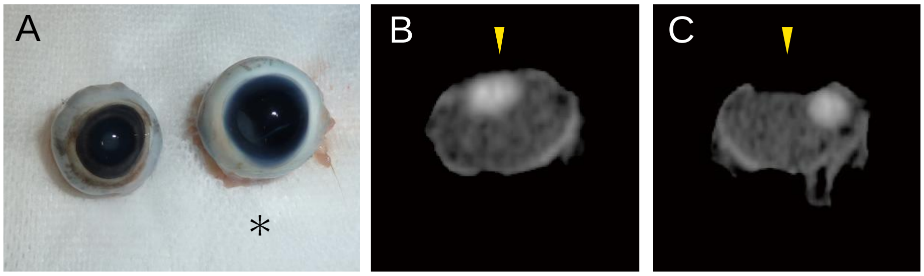

Figure S6. Ectopia lentis appeared in a homozygous *FBN1* mutant piglet.

(**A**) Eyes of a homozygous *FBN1* mutant piglet (*) and WT piglet (left). (**B**, **C**) CT image of the mutant pig eye (**C**) showed conspicuous dislocation of the lens (white circular portion), compared to the eye of a WT sibling (**B**). Arrowhead: cornea; white circular portion: lens.

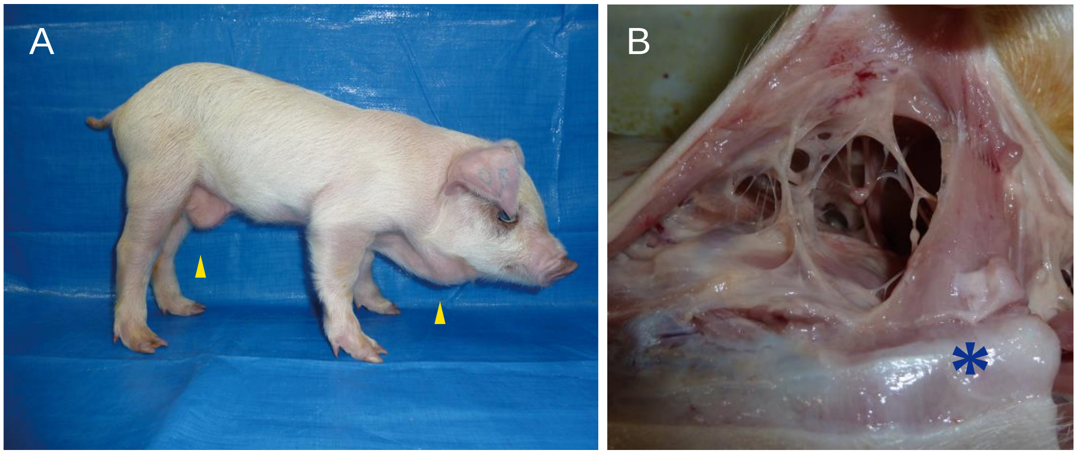

Figure S7. Lipodystrophy of a homozygous *FBN1* mutant piglet.

(**A**) Appearance of a homozygous *FBN1* mutant piglet with sagging skin of the front neck and abdomen (arrow head). (**B**) Skin incision of a homozygous *FBN1* mutant piglet revealed that subcutaneous adipose tissue of the chest, front neck, axilla and lower abdomen was underdeveloped and the adipose tissues was connected very loosely to other subcutaneous tissue, forming cavities. The asterisk indicates the sternum.

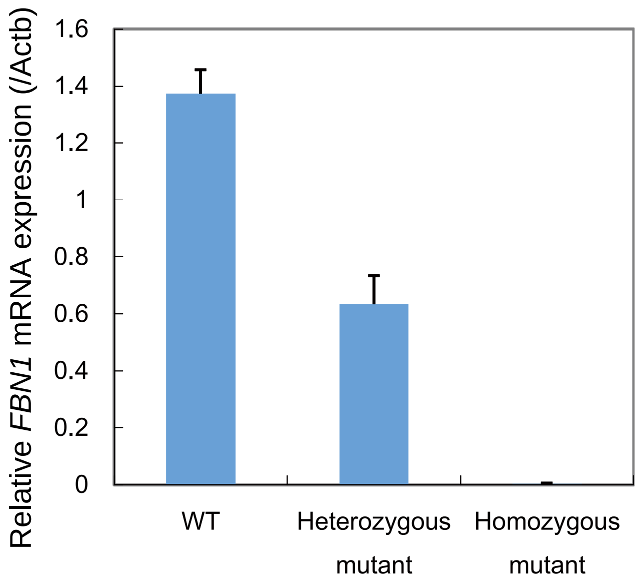

Figure S8. qRT-PCR analysis of *FBN1* expression in fibroblast cells from *FBN1* mutant piglets. Data obtained from three replicated experiments. The data are presented as the mean ± SD.

Supplementary methods

Analysis of ectopia lentis

Noncontrast enhanced-CT scans of formalin-fixed eyeballs were performed using a 4-row helical CT scanner (ECLOS, Hitachi Medical Corporation, Tokyo, Japan). The scan settings were as follows: 120 kVp, 100 mA, and section thickness of 0.625 mm.

Real-time expression analysis

Quantitative real-time PCR (qRT-PCR) was performed to evaluate FBN1 mRNA expression in tail-tip fibroblast cells established from WT, heterozygous mutant, and homozygous mutant pigs. RNA was isolated from the cells by using RNeasy Plus Micro and Mini Kits (QIAGEN). First-strand cDNA was synthesized using SuperScript III (Life Technologies) and random hexamers. qRT-PCR reactions were performed using StepOne Plus Real-Time PCR System (Life Technologies) with Premix Ex Taq (Probe qPCR) (TaKaRa Bio), TaqMan probe (Takara Bio) for FBN1: 5′-CGACCACCAGTGGAATATCCATAT and primers for FBN1 were 5′-GACCGCAAATTCCAGTC and 5′-TAATCAGTGACGTTGACAG, whereas those for the internal control, beta-actin (Actb), were 5′-TCCTTCCTGATGTCCACGTCGCACTTC (TaqMan probe), 5′-GCCCTCCTTCTTGGGCATG and 5′-CAGCACCGTGTTGGCGTAG. The ΔΔCT-method was used to determine the relative expression normalized to Actb expression. This experiment was performed three times and the average results were reported.
